# AI-Enhanced 3D Transperineal Ultrasound: Advancing Biometric Measurements for Precise Prolapse Severity Assessment

**DOI:** 10.3390/bioengineering12070754

**Published:** 2025-07-11

**Authors:** Desirèe De Vicari, Marta Barba, Alice Cola, Clarissa Costa, Mariachiara Palucci, Matteo Frigerio

**Affiliations:** Department of Gynecology, IRCCS San Gerardo dei Tintori, University of Milano-Bicocca, 20900 Monza, Italy; m.barba8792@gmail.com (M.B.); alice.cola1@gmail.com (A.C.); c.costa14@campus.unimib.it (C.C.); m.palucci1@campus.unimib.it (M.P.)

**Keywords:** pelvic organ prolapse, transperineal 3D ultrasound, artificial intelligence

## Abstract

Pelvic organ prolapse (POP) is a common pelvic floor disorder with substantial impact on women’s quality of life, necessitating accurate and reproducible diagnostic methods. This study investigates the use of three-dimensional (3D) transperineal ultrasound, integrated with artificial intelligence (AI), to evaluate pelvic floor biomechanics and identify correlations between biometric parameters and prolapse severity. Thirty-seven female patients diagnosed with genital prolapse (mean age: 65.3 ± 10.6 years; mean BMI: 29.5 ± 3.8) were enrolled. All participants underwent standardized 3D transperineal ultrasound using the Mindray Smart Pelvic system, an AI-assisted imaging platform. Key biometric parameters—anteroposterior diameter, laterolateral diameter, and genital hiatus area—were measured under three functional states: rest, maximal Valsalva maneuver, and voluntary pelvic floor contraction. Additionally, two functional indices were derived: the distensibility index (ratio of Valsalva to rest) and the contractility index (ratio of contraction to rest), reflecting pelvic floor elasticity and muscular function, respectively. Statistical analysis included descriptive statistics and univariate correlation analysis using Pelvic Organ Prolapse Quantification (POP-Q) system scores. Results revealed a significant correlation between laterolateral diameter and prolapse severity across multiple compartments and functional states. In apical prolapse, the laterolateral diameter measured at rest and during both Valsalva and contraction showed positive correlations with POP-Q point C, indicating increasing transverse pelvic dimensions with more advanced prolapse (e.g., r = 0.42 to 0.58; *p* < 0.05). In anterior compartment prolapse, the same parameter measured during Valsalva and contraction correlated significantly with POP-Q point AA (e.g., r = 0.45 to 0.61; *p* < 0.05). Anteroposterior diameters and genital hiatus area were also analyzed but showed weaker or inconsistent correlations. AI integration facilitated real-time image segmentation and automated measurement, reducing operator dependency and increasing reproducibility. These findings highlight the laterolateral diameter as a strong, reproducible anatomical marker for POP severity, particularly when assessed dynamically. The combined use of AI-enhanced imaging and functional indices provides a novel, standardized, and objective approach for assessing pelvic floor dysfunction. This methodology supports more accurate diagnosis, individualized management planning, and long-term monitoring of pelvic floor disorders.

## 1. Introduction

Transperineal ultrasound is a fundamental diagnostic tool in the assessment of pelvic floor dysfunctions, offering a non-invasive, cost-effective, and dynamic approach to evaluating pelvic organ support and function. Over the past two decades, significant advancements in imaging technology have enhanced the clinical utility of ultrasound, particularly with the development of three-dimensional (3D) and four-dimensional (4D) imaging modalities [1,2,3]. These innovations have revolutionized the anatomical and functional evaluation of the pelvic floor, allowing for more accurate, detailed, and reproducible assessments while reducing operator dependency compared to traditional two-dimensional methods [4]. As a result, transperineal ultrasound has become an essential component in the diagnosis and management of pelvic organ prolapse (POP), stress urinary incontinence (SUI), and levator ani muscle injuries [5,6].

### 1.1. Ultrasound Diagnostics in Urogynecology

The application of ultrasound in urogynecological diagnostics enables real-time visualization and assessment of pelvic floor structures. This imaging technique is particularly valuable for evaluating functional maneuvers such as the Valsalva maneuver and pelvic floor muscle contraction, providing a detailed, dynamic view of anatomical relationships and dysfunctions [1]. Unlike static imaging modalities like MRI, ultrasound allows for the direct observation of pelvic floor behavior under physiological conditions, improving the ability to detect abnormalities such as bladder neck descent, levator ani muscle avulsion, and excessive levator hiatus distensibility [2,7,8].

The introduction of 3D and 4D ultrasound imaging has significantly expanded diagnostic capabilities. These technologies facilitate the volumetric assessment of pelvic structures, enabling precise evaluation of morphological defects such as levator ani muscle trauma, excessive tissue laxity, and connective tissue insufficiency—key factors implicated in the pathogenesis of pelvic organ prolapse [4,9,10]. Moreover, 3D ultrasound allows for tomographic imaging, where multiple planes can be analyzed simultaneously, enhancing the detection of subtle structural abnormalities that might be overlooked using conventional 2D imaging.

### 1.2. Ultrasound Risk Factors for Prolapse Recurrence

Ultrasound imaging has played a pivotal role in identifying risk factors that predispose individuals to prolapse recurrence after surgical or conservative management. Several biomechanical and anatomical characteristics have been associated with an increased risk of recurrent pelvic organ prolapse, including the following:

- Levator Ani Muscle Avulsion: A lesion commonly observed in patients with a history of vaginal delivery, characterized by the detachment of the puborectalis muscle from the pubic ramus. This injury, which occurs in approximately 10–35% of women who have undergone vaginal childbirth, has been strongly linked to anterior and central compartment prolapse. It is now recognized as a significant predictor of postoperative prolapse recurrence, as loss of levator integrity results in diminished pelvic floor support [6,11,12].
- Levator Hiatus Overdistension: Defined as an excessive enlargement of the levator hiatus (≥25 cm^2^ during the Valsalva maneuver), this condition is a major contributor to both primary and recurrent prolapse. Overdistension of the hiatus weakens pelvic support structures, making women more susceptible to recurrent pelvic organ descent despite surgical intervention [4,13,14].

These ultrasound-identified factors, combined with clinical variables such as parity, neonatal birth weight, prolonged second-stage labor, operative delivery, and a history of complicated vaginal birth, should be carefully evaluated when determining the optimal management strategy for patients with pelvic floor disorders [15,16]. A personalized approach that incorporates both imaging-based risk assessment and individual patient characteristics is essential for improving long-term treatment outcomes.

### 1.3. Implementation of Artificial Intelligence in Ultrasound Imaging

Artificial intelligence (AI) is transforming the landscape of diagnostic imaging in urogynecology, offering powerful tools to enhance accuracy, efficiency, and reproducibility. Advanced machine learning and deep learning algorithms can analyze vast amounts of ultrasound data, identify subtle patterns that may be challenging for the human eye to detect, and provide automated measurements with high precision [17]. The integration of AI in ultrasound technology is particularly valuable for pelvic floor imaging, where complex anatomical structures and dynamic functional assessments require detailed interpretation.

A significant breakthrough in AI-driven ultrasound diagnostics has been the development of automated systems for quantifying levator hiatus dimensions and detecting levator ani muscle avulsions through tomographic ultrasound imaging [17,18]. These AI-enhanced systems help to reduce inter-operator variability, improve measurement consistency, and optimize clinical decision-making by providing standardized assessments of pelvic floor integrity [19]. In particular, AI-powered image segmentation algorithms allow for rapid and accurate delineation of soft tissue structures, facilitating more reliable detection of prolapse-related abnormalities.

### 1.4. The Mindray Technology: AI-Driven Advancements in Pelvic Floor Ultrasound

Among the cutting-edge technologies available today, Mindray has pioneered innovative solutions aimed at enhancing the diagnostic capabilities of ultrasound in pelvic floor disorders. The Mindray Smart Pelvic system, an AI-integrated ultrasound platform, represents a significant advancement in urogynecological imaging. This technology incorporates advanced machine learning algorithms to automatically identify and quantify key risk factors for prolapse, such as levator ani muscle avulsion and levator hiatus over-distension. Specifically, the core of the Smart Pelvic system relies on a multi-stage hybrid deep learning architecture, which integrates convolutional neural networks (CNNs) for image segmentation with rule-based geometric models for anatomical alignment. The system begins with image preprocessing, which identifies key anatomical structures in the pelvic floor volume data—including the symphysis pubis, urethra, anal canal, and anorectal angle. These landmarks are used to standardize the volume orientation through rule-based geometric alignment, ensuring that structures such as the posterior border of the pubic symphysis and the anorectal angle are aligned in a reproducible axial plane across patients.

Following this, a deep learning-based segmentation algorithm, built on a modified U-Net architecture optimized for 3D/4D ultrasound imaging, is applied. The U-Net model is trained on a proprietary, anonymized dataset of over 1000 manually labeled clinical pelvic ultrasound volumes, annotated by experienced radiologists. This network automatically delineates the minimum levator hiatus (LH) section and calculates key quantitative parameters, including area, circumference (Circ), anteroposterior diameter (AP Diam), and transverse diameter (Lateral Diam), all with sub-second inference times. A lightweight CNN-based landmark detection module precedes the segmentation step, facilitating consistent anatomical localization and enhancing robustness across variable input quality.

Complementing the axial segmentation pipeline, a dedicated multi-plane tomographic analysis algorithm, powered by Mindray’s iPage engine, enables automated slice acquisition for evaluating the continuity of the levator ani muscle. This algorithm defines cross-sectional planes at regular intervals perpendicular to the pelvic floor, providing consistent tomographic visualization of the muscle and facilitating measurements. All segmentation outputs remain editable post-processing, offering clinicians both the efficiency of automation and the flexibility of manual refinement.

The performance of these algorithms has undergone rigorous internal and external validation. In a clinical study conducted at the Second People’s Hospital of Shenzhen, 119 patient datasets across varying cystocele grades (0–III) were analyzed to compare measurement efficiency and diagnostic agreement between junior clinicians (Group D1), senior clinicians (Group D2), and the Smart Pelvic AI tool (Group D3). Time consumption for LH measurements showed a dramatic reduction, with the AI group averaging only ~2.7 s per case, compared to 78–91 s in Group D1 and 60–65 s in Group D2. Coincidence rates between AI-derived LH contours and expert manual annotations exceeded 95% across all prolapse grades. Notably, the system achieved a peak coincidence rate of 96.35% when benchmarked against expert-drawn LH contours in 100 Valsalva cases analyzed at the Third Affiliated Hospital of Sun Yat-Sen University, indicating excellent agreement and diagnostic reproducibility [19].

Beyond segmentation, the platform’s [multi-plane mode] employs automated tomography through iPage sequencing to visualize levator ani muscle continuity, allowing fast identification of avulsion injuries. Levator urethral gap (LUG) is also automatically computed bilaterally to support quantitative assessment of muscle detachment. Additionally, the anal sphincter positioning module applies volumetric reconstruction and layer-wise tomographic analysis based on anatomical alignment to ensure consistent cross-sectional assessment.

By streamlining the diagnostic workflow and reducing operator-dependent variability, Mindray’s AI-enhanced system not only enhances productivity but also improves diagnostic objectivity, accuracy, and reproducibility in the evaluation of pelvic floor dysfunction [20,21].

The use of AI-driven analysis tools offers several clinical advantages, including:

- Enhanced Detection of Pelvic Floor Abnormalities: Automated quantification of levator hiatus dimensions and prolapse-related changes improves diagnostic sensitivity and specificity.
- Reduced Variability in Interpretation: Standardized AI-based measurements minimize inter-observer differences, ensuring consistency across different examiners and clinical settings.
- Optimized Treatment Planning: By providing more precise and objective assessments of pelvic floor biomechanics, AI-integrated ultrasound technology facilitates personalized treatment strategies, including surgical planning and rehabilitation programs.

As the field of urogynecology continues to evolve, the integration of AI into 3D transperineal ultrasound represents a promising step forward in improving the diagnosis and management of pelvic floor disorders. Future research should focus on validating these AI-driven approaches in larger, multicenter patient cohorts, investigating their performance across diverse anatomical presentations, and exploring their potential role in predicting long-term treatment outcomes. By harnessing the power of artificial intelligence, clinicians can enhance the accuracy, efficiency, and accessibility of pelvic floor imaging, ultimately leading to better patient care and improved clinical outcomes.

### 1.5. Aim of the Study

The primary objective of this study is to assess the correlation between the severity of pelvic organ prolapse (POP) and pelvic floor biometric parameters obtained through three-dimensional (3D) transperineal ultrasound imaging. By integrating artificial intelligence (AI) into the standard ultrasound examination, we aim to enhance the precision, reproducibility, and clinical utility of pelvic floor assessments.

This study aims to explore the correlation between the severity of pelvic organ prolapse and key pelvic floor biometric parameters obtained through three-dimensional transperineal ultrasound imaging. Particular attention is given to the assessment of anteroposterior diameter, laterolateral diameter, and genital hiatus area, examining their variations across different functional conditions, including rest, the Valsalva maneuver, and voluntary pelvic floor muscle contraction.

Moreover, this research seeks to determine the extent to which artificial intelligence-enhanced imaging can contribute to reducing measurement variability and improving diagnostic accuracy, particularly in the identification of subtle alterations in pelvic support structures. The potential implications of these advancements in clinical decision-making are also considered, with a focus on risk stratification, individualized treatment planning, and prognosis prediction for patients presenting with varying degrees of prolapse severity.

By integrating AI-based automated analysis with conventional ultrasound imaging, this study aims to bridge the gap between operator-dependent measurements and objective, reproducible quantifications, ultimately advancing the diagnostic and therapeutic management of pelvic organ prolapse.

## 2. Materials and Methods

This cross-sectional study included a total of 37 female patients diagnosed with symptomatic pelvic organ prolapse. The inclusion criteria were as follows: (1) clinically confirmed genital prolapse based on the Pelvic Organ Prolapse Quantification (POP-Q) system; (2) no prior pelvic reconstructive surgery; and (3) the ability to perform Valsalva and voluntary pelvic floor muscle contractions. Exclusion criteria included pregnancy, neurological disorders affecting pelvic floor function, and previous prolapse surgery.

### 2.1. Patient Population

The study cohort consisted of women aged between 34 and 80 years, with a mean age of 65.3 years (standard deviation: 10.6). The mean body mass index (BMI) was 29.5 kg/m^2^ (SD: 3.8). The average parity was 1.81, with three patients being nulliparous, eight having had a single vaginal delivery, and the remainder having experienced two or more vaginal births (Table 1).

In terms of prolapse severity, the most commonly affected compartment was the anterior compartment, with a mean severity score of 2.9, followed by the apical compartment (mean: 2.86) and the posterior compartment (mean: 1.3).

### 2.2. Ultrasound Examination Protocol

All patients underwent a standardized 3D perineal ultrasound evaluation using a Mindray ultrasound system (Nuewa I9, Mindray, Shenzhen, China) equipped with artificial intelligence (Mindray Smart Pelvic). The examinations were performed with patients in the semi-recumbent lithotomy position, with an empty bladder to minimize confounding effects on pelvic organ mobility. The transperineal probe was placed over the perineum with minimal pressure to avoid distortion of the pelvic floor structures.

The AI-enhanced ultrasound system enabled fully automated acquisition, segmentation, and quantification of pelvic floor structures, delivering high-resolution volumetric data with minimal operator intervention. Notably, no manual corrections to the AI-generated outputs were necessary, ensuring that all measurements were conducted in a completely automated manner.

Measurements included the following:

- Anteroposterior (AP) diameter
- Laterolateral (LL) diameter
- Genital hiatus area

Each parameter was recorded in the following three distinct functional conditions:

1. At rest (R)—baseline pelvic floor measurements with no voluntary muscle activity.
2. During Valsalva maneuver (V)—assessment under maximal abdominal pressure, simulating prolapse descent.
3. During contraction (S)—evaluation of voluntary pelvic floor muscle contraction (Table 2).

The data collected were analyzed using descriptive statistics to determine mean values and standard deviations for each measurement. Univariate correlation analyses were conducted to explore associations between prolapse severity and biometric parameters.

The methodology employed in this study was designed to ensure a comprehensive and accurate assessment of pelvic floor dysfunction, particularly focusing on the severity of pelvic organ prolapse. One of the key innovations in this research was the use of AI-assisted measurements through the Mindray Smart Pelvic AI module. This advanced technology played a crucial role in automating the data processing, which helped to minimize inter-operator variability—a common issue in ultrasound diagnostics. By utilizing AI algorithms, the system was able to accurately delineate the levator hiatus and identify key anatomical landmarks, which significantly reduced the subjectivity involved in manual assessments. This allowed for a more consistent and reliable measurement process, ensuring the precision of the collected data and enhancing the overall robustness of the study.

In terms of statistical analysis, the data were processed using JMP 17 software (SAS Institute, Cary, NC, USA). To assess the relationships between different ultrasound parameters and the severity of prolapse, Pearson’s correlation coefficient was employed. A *p*-value of less than 0.05 was considered statistically significant, ensuring that the correlations observed were not due to chance. Furthermore, additional regression analyses were conducted to identify independent predictors of prolapse severity. This helped to determine which specific variables were most strongly associated with the progression of prolapse, providing valuable insights into the underlying factors that contribute to pelvic floor dysfunction.

Ethical considerations were also a critical aspect of the study. Prior to enrollment, the study received approval from the institutional review board (IRB), ensuring that it adhered to the ethical standards required for clinical research. All participants were fully informed about the study’s objectives and procedures, and their consent was obtained before participation.

## 3. Results

The analysis revealed significant correlations between laterolateral diameter and the severity of pelvic organ prolapse. These correlations were consistently observed across all three functional conditions: at rest, during the Valsalva maneuver, and during contraction.

For apical prolapse, a statistically significant association was found between prolapse severity and transverse diameter at rest, with a positive correlation coefficient indicating that larger laterolateral dimensions were associated with more severe prolapse. Similar correlations were noted during the Valsalva maneuver and contraction, reinforcing the role of transverse diameter as a reliable parameter in assessing prolapse severity.

In the case of anterior compartment prolapse, significant correlations were identified between laterolateral diameter during the Valsalva maneuver and anatomical reference points used in the POP-Q system, specifically Points AA and C. Additionally, transverse diameter during contraction was significantly associated with these same reference points, suggesting that this parameter may be a key determinant in anterior compartment support.

Although absolute pelvic floor measurements demonstrated clear relationships with prolapse severity, the analysis of functional indices yielded more nuanced findings. Distensibility and contractility ratios, while providing valuable insights into pelvic floor biomechanics, did not show statistically significant correlations with prolapse severity within the study population. However, these ratios remain clinically relevant as potential predictors of pelvic floor dysfunction and warrant further investigation in larger cohorts.

## 4. Discussion

This study underscores the growing relevance of three-dimensional (3D) ultrasound in the assessment of pelvic organ prolapse [1,2]. Among the parameters analyzed, the laterolateral diameter demonstrated a statistically significant and independent association with prolapse severity, highlighting its potential clinical utility [3,4]. Notably, this correlation was observed independently of levator ani muscle avulsion, suggesting that laterolateral diameter may serve as a standalone diagnostic marker in the evaluation of pelvic floor dysfunction [5,6].

Given the well-established relationship between levator ani avulsion and prolapse severity [6,7], the independent predictive value of the transverse diameter is particularly noteworthy. This parameter could represent an additional, easily measurable marker for risk stratification, thereby contributing to earlier identification of high-risk patients and more tailored treatment planning [8,9].

Nonetheless, several limitations should be considered when interpreting the findings. The study cohort consisted of only 37 participants, which limits the statistical power and generalizability of the results. However, in early-phase diagnostic investigations focused on imaging-based parameter development, such sample sizes are acceptable, particularly when advanced imaging protocols and standardized measurements are employed to reduce variability.

In addition, the study population exhibited considerable heterogeneity in terms of age, body mass index (BMI), and parity. These factors are known to influence pelvic floor anatomy and function and may act as potential confounders. While this variability limits internal validity, it also enhances the external applicability of the findings to routine clinical settings. Stratified analyses or multivariate adjustments were not performed due to the limited sample size, representing a further analytical constraint. Future studies with larger, more homogeneous cohorts are warranted to control for these confounding variables and validate the observed associations [10,11,12,13,14].

Another important limitation is the absence of a control or comparator group. Without a conventional imaging cohort for reference, the added diagnostic value of artificial intelligence (AI)-enhanced ultrasound could not be directly assessed. While this was not the primary objective of the present study, future research should aim to compare AI-assisted and manual approaches to quantify improvements in diagnostic performance, reproducibility, and clinical efficiency.

The analytical approach employed relied exclusively on univariate correlations, with no multivariate models applied to account for potential confounders such as BMI, parity, or age. This limits the interpretability of the associations observed and highlights the need for more complex statistical modeling in future work.

Regarding functional assessment, parameters such as distensibility and contractility ratios did not show significant correlations with prolapse severity. Although these findings were briefly mentioned, they deserve further attention. The lack of association may reflect true physiological dissociation between structural and functional impairments or could be influenced by variability in patient compliance during dynamic maneuvers and limitations in ultrasound-based functional quantification. These indices may still be clinically relevant in longitudinal follow-up or postoperative evaluation and merit further investigation.

### Future Perspectives

Despite the aforementioned limitations, the findings support the potential role of laterolateral diameter as a practical imaging biomarker for prolapse evaluation. Future research should explore its prognostic value in predicting disease progression, response to therapy, and recurrence rates [10,12,15].

The integration of AI in pelvic floor ultrasound represents a major innovation. AI algorithms offer the ability to automate complex measurements, reduce operator dependence, and improve consistency across clinical settings [17,18]. However, the present study provides limited detail regarding the AI methodology, including algorithm architecture, training dataset characteristics, and validation metrics. For clinical adoption, future studies should adhere to transparent reporting standards that clearly define AI performance and generalizability.

If adequately validated, AI-enhanced ultrasound could facilitate more accurate, efficient, and standardized assessments of pelvic floor anatomy and function. Such tools have the potential to improve risk stratification, support personalized treatment planning, and enhance follow-up care for patients with pelvic organ prolapse [20,21]. Ultimately, the convergence of advanced imaging and artificial intelligence may contribute to more precise, data-driven urogynecological practice [17,18,20,21].

## 5. Conclusions

This study underscores the value of three-dimensional transperineal ultrasound, particularly in combination with artificial intelligence, for the objective assessment of pelvic organ prolapse [1,17]. Among the parameters analyzed, laterolateral diameter emerged as a key biometric marker, demonstrating a significant correlation with prolapse severity across different functional conditions [3,5]. This finding highlights the importance of advanced imaging techniques in improving the accuracy and reliability of urogynecological evaluations [2,9].

The role of AI-enhanced ultrasound in reducing operator dependency and increasing measurement reproducibility represents a major advancement in the field [18,20,21]. By automating the identification of key anatomical features and improving diagnostic consistency, AI-driven approaches have the potential to refine risk stratification, optimize treatment planning, and contribute to a more personalized approach in managing pelvic floor disorders [17,20].

Although functional indices such as distensibility and contractility ratios did not show statistically significant correlations with prolapse severity in this study, they remain promising parameters for future research [10,13]. A more comprehensive understanding of pelvic floor biomechanics, particularly in larger and more homogeneous cohorts, could further enhance clinical decision-making and improve patient outcomes [14,15,16].

Future studies should focus on expanding the dataset and integrating both structural and functional analyses to develop predictive models for prolapse progression and treatment success. The continued refinement of AI-assisted ultrasound techniques will likely play a pivotal role in advancing the field of urogynecology, offering clinicians more precise and efficient tools for diagnosing and managing pelvic floor dysfunctions.

## Figures and Tables

**Table 1:** Population characteristics.

Variable	Mean	Range
BMI (kg/m^2^)	29.5	21–42
Age (years)	65.3	34–80
Parity (n)	1.81	0–4

**Table 2:** Mean and SD of levator hiatus measurements calculated using AI-enhanced 3D perineal ultrasound at rest (R), during Valsalva (V), and during contraction (S).

	Mean Area cm^2^ (DS)	Mean AP Diameter mm (DS)	Mean LL Diameter mm (DS)
At rest (R)	26.27 (7.61)	65.51 (7.7)	55.2 (10.23)
Valsalva (V)	31.8 (9.4)	71.7 (10.3)	55.3 (9.7)
Contraction (S)	21.4 (6.5)	56.3 (9.3)	48.1 (9.5)

## Data Availability

The original contributions presented in this study are included in the article. Further inquiries can be directed to the corresponding authors.
